# An Overview of Strobilurin Fungicide Degradation:Current Status and Future Perspective

**DOI:** 10.3389/fmicb.2020.00389

**Published:** 2020-03-12

**Authors:** Yanmei Feng, Yaohua Huang, Hui Zhan, Pankaj Bhatt, Shaohua Chen

**Affiliations:** ^1^State Key Laboratory for Conservation and Utilization of Subtropical Agro-bioresources, Guangdong Laboratory for Lingnan Modern Agriculture, Integrative Microbiology Research Centre, South China Agricultural University, Guangzhou, China; ^2^Guangdong Province Key Laboratory of Microbial Signals and Disease Control, Guangzhou, China

**Keywords:** strobilurin, ecotoxicity, biodegradation, bioremediation, transformation, degradation pathways

## Abstract

Strobilurin fungicides have been widely used in agricultural fields for decades. These pesticides are designed to manage fungal pathogens, although their broad-spectrum mode of action also produces non-target impacts. Therefore, the removal of strobilurins from ecosystems has received much attention. Different remediation technologies have been developed to eliminate pesticide residues from soil/water environments, such as photodecomposition, ozonation, adsorption, incineration, and biodegradation. Compared with conventional methods, bioremediation is considered a cost-effective and ecofriendly approach for the removal of pesticide residues. Several strobilurin-degrading microbes and microbial communities have been reported to effectively utilize pesticide residues as a carbon and nitrogen source. The degradation pathways of strobilurins and the fate of several metabolites have been reported. Further in-depth studies based on molecular biology and genetics are needed to elaborate their role in the evolution of novel catabolic pathways and the microbial degradation of strobilurins. The present review summarizes recent progress in strobilurin degradation and comprehensively discusses the potential of strobilurin-degrading microorganisms in the bioremediation of contaminated environments.

## Introduction

**FIGURE 1:**
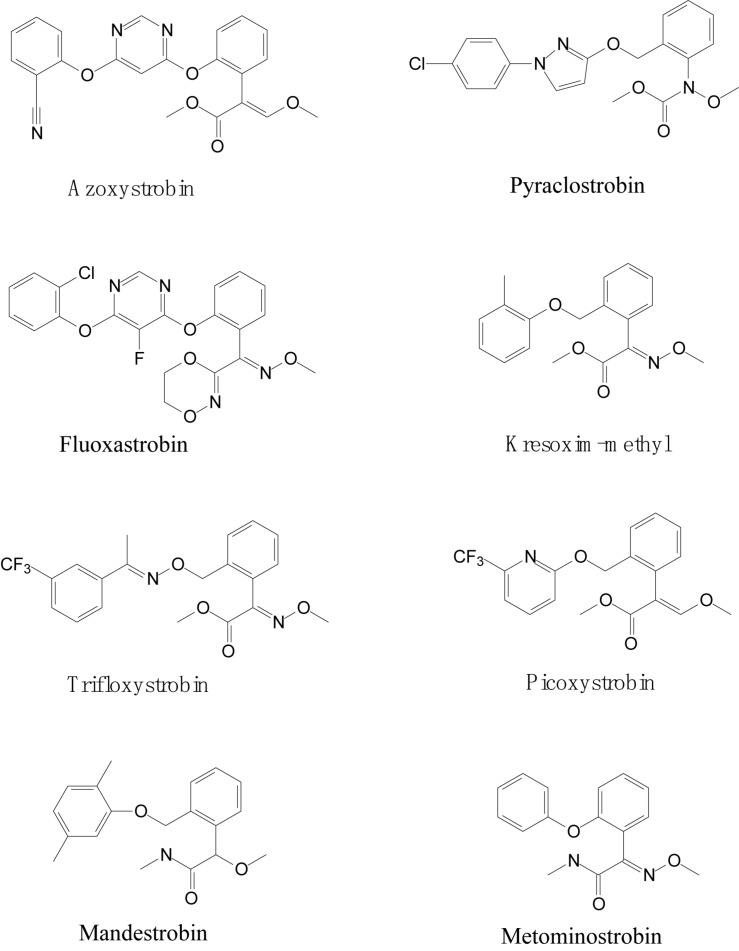
Molecular structures of strobilurins.

Strobilurin fungicides are globally used to combat white mold, rot, early and late leaf spot, rusts and rice blast (Bartett et al., 2001; FAO Meeting, 2008). Mushrooms (*Basidiomycetes*) are natural sources of strobilurins, and the first natural strobilurin compound, Strobilurin-A, was originally isolated from the mushroom *Strobilurus tenacellus* by Anke et al. (1977). The first patent for a strobilurin fungicide (azoxystrobin) was introduced in the German market in 1996 (Sauter et al., 1999; Bartett et al., 2001). Subsequently, a series of strobilurin fungicides, including azoxystrobin, pyraclostrobin, fluoxastrobin, kresoxim-methyl, trifloxystrobin, picoxystrobin, mandestrobin, and metominostrobin, were developed and marketed (Rodrigues et al., 2013; Khandelwal et al., 2014). Structurally, the presence of toxiphoric (E)-β-methoxyacrylate group is the main feature of strobilurin fungicides (Balba, 2007), as presented in Figure 1. Strobilurin fungicides are also referred as Q_o_I fungicides because of their unique mechanism of action. They specifically bind to the quinol oxidation (Q_o_) site of cytochrome b to inhibit mitochondrial respiration. This binding blocks electron transfer between cytochrome b and cytochrome c1 and inhibits the synthesis of nicotinamide adenine dinucleotide (NADH) oxidation and the mitochondrial membrane protein adenosine triphosphate (ATP) (Hnátová et al., 2003; Isamu and Makoto, 2005; Balba, 2007; Rodrigues et al., 2013). The fungicidal action of strobilurins is novel and non-target specific.

Strobilurins present broad-spectrum, rapid and highly efficient germicidal activities, are cost effective and rapidly degrade during plant metabolism, and these benefits have contributed to the large-scale use of these fungicides. However, environmental contamination and non-target toxicity due to the long-term use of strobilurins has raised serious public health concerns. For instance, according to the European Food Safety Authority (EFSA), azoxystrobin is frequently found in foodstuffs (EFSA, 2010). Environmental concentrations of azoxystrobin higher than the Regulatory Acceptable Concentration (RAC) have been found in ecosystems, which poses a serious risk to soil organisms, aquatic organisms, and mammals (Warming et al., 2009; Dijksterhuis et al., 2011; Liu et al., 2013; Mostafalou and Abdollahi, 2013; Rodrigues et al., 2013; Regueiro et al., 2015; Li et al., 2016; Pearson et al., 2016; Zubrod et al., 2019). In addition, strobilurins are susceptible to resistance because they act on one specific site of fungal pathogens. Several resistance genes from strobilurin-treated fungi have been reported (Zheng and Köeller, 1997; Fisher et al., 2004; Walker et al., 2009; Piszczek et al., 2017). These reports suggested that strobilurins can potentially cause long-term adverse effects to the ecosystem.

Strobilurins can be degraded either through biotic or abiotic approaches, such as incineration, photodecomposition, adsorption, and biodegradation. Compared with physicochemical methods, the microbial degradation of pesticide residues is emerging as an efficient “green” strategy (Chen et al., 2011; Harms et al., 2011; Xiao et al., 2015; Arora et al., 2017; Cycoń et al., 2017; Yang et al., 2018). Several reports have focused on the isolation and characterization of strobilurin-degrading microbes and microbial communities (Lopes et al., 2010; Clinton et al., 2011; Howell et al., 2014; Bacmaga et al., 2015; Chen et al., 2018). These microbes include *Bacillus*, *Pseudomonas*, *Klebsiella*, *Stenotrophomonas*, *Arthrobacter*, *Rhodanobacter*, *Cupriavidus*, and *Aphanoascus.* The metabolic pathways of strobilurins and the fate of several metabolites have been reported. However, there is a limited number of studies on strobilurin-degrading enzymes and the corresponding genes in microorganisms. In addition, few reviews have focused on the mechanisms and degradation pathways of strobilurins. In this review, we aim to summarize strobilurin degradation mechanisms and analyze the bioremediation potential of strobilurin-degrading microorganisms in contaminated soil/water environments.

## Physicochemical Transformation of Strobilurins

Strobilurin residues remain in the air, soil, or water after field applications, and the physicochemical properties of the associated environment affect the behavior and distribution of these residues (Rodrigues et al., 2013). Pesticide interacts with organic or mineral constituents as it reaches the soil and undergoes chemical and biological transformation (Bending et al., 2006). Generally, strobilurin compounds easily degrade in plants, animals, soil, and water (Lee et al., 1999; Balba, 2007). Joseph (1999) explored the abiotic degradation of azoxystrobin in three different soils and reported that azoxystrobin was photodegraded at a half-life (*t*_1__/__2_) of less than 14 days under field conditions while the half-life of azoxystrobin was approximately 8–12 weeks under dark aerobic conditions. The study also revealed that hydrolysis of the ester moiety is the major metabolic pathway of azoxystrobin. Boudina et al. (2007) studied the photochemical behavior of azoxystrobin in aqueous solutions and revealed that multiple parallel reaction pathways occur during its phototransformation. These pathways include photoisomerization (*E*→*Z*), acrylate double bond cleavage, photohydrolysis of nitrile group and methyl ester, and phenol and oxidative cleavage of acrylate double bonds after the cleavage of aromatic rings. The results also showed that azoxystrobin absorbs light at higher wavelengths (290 nm) in aqueous environments, which facilitate its photodegradation. Singh et al. (2010) explored the metabolism of (14) C-azoxstrobin under aqueous conditions at pH 4, 7, and 9 and identified metabolite R234886 as (E)-2-{2-[6-(2-cyano-phenoxy) pyrimidin-4-yloxy] phenyl}-3-methoxyacrylic acid, which is the main metabolite of azoxystrobin. Compared to acidic (pH 4) or neutral (pH 7) conditions, metabolite formation occurred in larger quantities and comparatively faster under alkaline (pH 9) conditions. Chastain et al. (2013) found that the photochemical reactivity of azoxystrobin was enhanced as the solvent polarity decreased. This phenomenon indicates that the accumulation of azoxystrobin tends to occur inside the cuticle, where it is photodegraded, or at the surface of crop leaves.

Azoxystrobin metabolism is similar to the degradation of methoxyiminoacetate (Balba, 2007) and the fate of trifloxystrobin in plants and kresoxim-methyl in soils, plants, and rats. Khandelwal et al. (2016) investigated the persistence of kresoxim-methyl at different temperatures, pH, atmospheric CO_2_ levels and light in aqueous conditions and revealed that it readily forms acid metabolites. The study emphasized that abiotic factors have significant effects on the dissipation of kresoxim-methyl under aqueous conditions. Similarly, photolysis has also been reported as the main degradation pathway of trifloxystrobin under field conditions, and the number of sunshine hours is the key influencing factor for the photolysis process (Wang et al., 2015). Trifloxystrobin residue was found in tomato, whereas its metabolite trifloxystrobin acid was observed in soil (Wang et al., 2014).

Reddy et al. (2013) reported that the organic matter content, microbial population and soil moisture affected the dissipation of pyraclostrobin, and their results showed the more rapid dissipation of pyraclostrobin under wet air conditions compared with dry conditions; moreover, the most rapid pyraclostrobin dissipation occurred in sludge-amended soil (*t*_1__/__2_ 9.2 days). Contrary to Reddy et al. (2013); Zeng et al. (2019) found that the degradation rate of pyraclostrobin was faster in aqueous solution under the UV photolysis reaction compared with that under sunlight. Unlike natural strobilurins that have the conventional methoxyacrylate or methoxyiminoacetate structure, mandestrobin possesses a unique methoxyacetamide moiety and showed resistance to alkaline hydrolysis (Adachi et al., 2018).

## Eco-Toxicity of Strobilurins

The excessive and long-term use of strobilurins has adversely affected ecosystems. Hydrolytically stable azoxystrobin presents low solubility (6.7 mg⋅L^–1^ at 20°C) at pH values between 4 and 9, which demonstrates its potential risk to water quality (Deb et al., 2010; EFSA, 2010; Singh et al., 2010). Many researchers have discovered that azoxystrobin is not only toxic to target fungi but also to non-target organisms. Friberg-Jensen et al. (2010) investigated the sub-lethal toxicity of azoxystrobin on different physiological parameters of egg-carrying *Daphnia magna* (such as the heart, filtering limbs, mandibles, and focal spine), and they concluded that after 24 h of activity, all response parameters decreased except the focal spine at an azoxystrobin concentration of 500 μg⋅L^–1^. DNA damage was observed in earthworms (*Eisenia fetida*) after azoxystrobin treatment (Han et al., 2014). The genotoxic effects of chronic and acute azoxystrobin concentrations in the erythrocytes of early life stages of brown trout *Salmo trutta fario* were assessed. The results highlighted the genotoxic threat to freshwater fish in azoxystrobin-contaminated rivers (Bony et al., 2008). Jia et al. (2018) also investigated the effects of azoxystrobin and picoxystrobin on the embryonic development and enzyme activity of zebrafish (*Danio rerio*). Their results indicated that both azoxystrobin and picoxystrobin caused dose- and time-dependent effects on embryonic development. Evaluations of the effects of azoxystrobin on biological activity in soil revealed that it changes microbial biodiversity by inhibiting the growth of organotrophic bacteria, actinomycetes, and fungi (Bacmaga et al., 2015).

Strobilurins were once considered less toxic to mammals (Bartlett et al., 2002); however, several authors have highlighted that scientific interpretations of their toxicity were limited because of gaps in the toxicological endpoints of fungicides (Battaglin et al., 2011). The toxicity of kresoxim-methyl and pyraclostrobin to the primary culture of mouse cortical neurons has been reported (Regueiro et al., 2015). *In vitro* studies have confirmed the genotoxicity and cytotoxicity of pyraclostrobin to human peripheral blood lymphocytes (Cayir et al., 2014). All of these studies suggested that the toxicity of strobilurins to organisms occurred via various routes.

## Possible Pathways in the Degradation of Strobilurins

**FIGURE 2:**
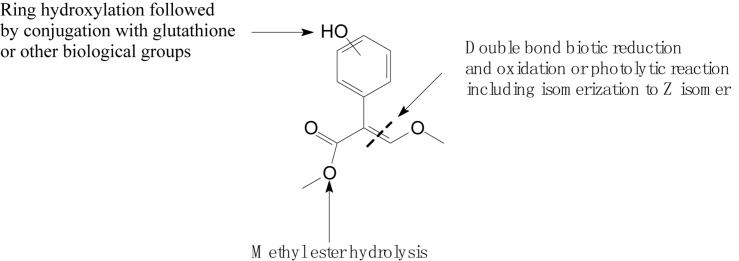
Basic degradation pathways of strobilurins (based on Balba, 2007).

**FIGURE 3:**
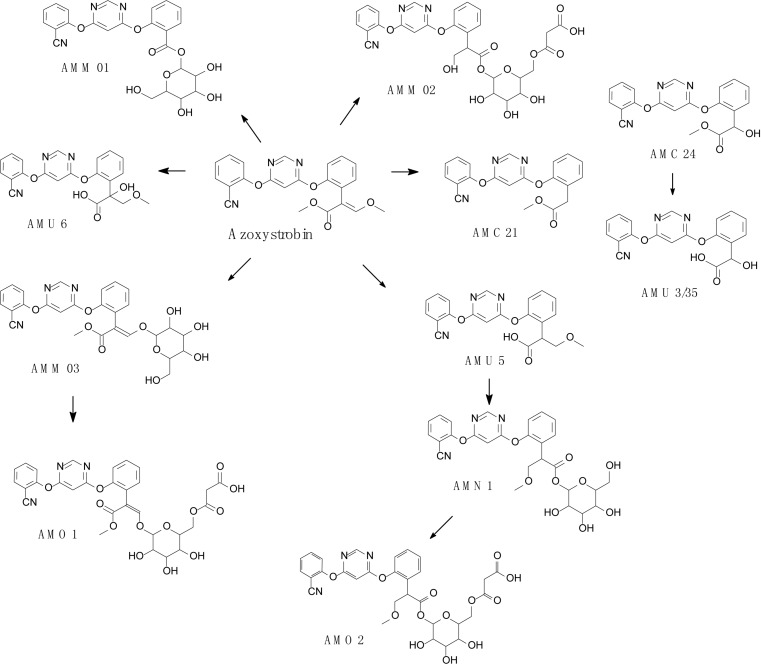
Proposed degradation pathway of azoxystrobin in *Brassica* species (based on Bauer et al., 2018).

**FIGURE 4:**
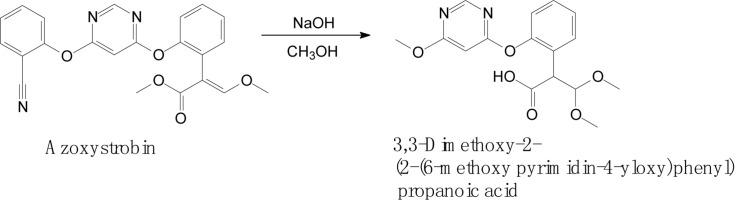
Proposed degradation pathway of azoxystrobin via the hydrolysis reaction (based on Zhang et al., 2011).

Complex structures of strobilurins provide several sites for metabolic reactions that follow multiple pathways. According to Balba (2007), the basic strobilurin degradation pathways involve methyl ester hydrolysis, ring hydroxylation followed by conjugation with glutathione or other biological groups, double bond biotic reduction and oxidation or photolytic reaction and isomerization to *Z* isomer (Figure 2). The double bond of strobilurins in the acrylic moiety was noted to be vulnerable to various degradation mechanisms. These interactions are possible between the amino acids of the enzyme’s active sites and the chemical bond of pesticides. Bauer et al. (2018) detected phase II metabolites of azoxystrobin in *Brassica* species by liquid chromatography and proposed its biotransformation pathway (Figure 3). They indicated that two main groups are separated in the initially activated phase I metabolites and conjugated phase II metabolites, and they were mostly formed via dealkylation reactions. Zhang et al. (2011) found an unexpected hydrolysis product, 3,3-dimethoxy-2-(2-(6-methoxy pyrimidin-4-yloxy)phenyl) propanoic acid, during the hydrolysis reaction of azoxystrobin in methanol. In this hydrolysis, the cyano-benzene ring in azoxystrobin is substituted by methoxy and an acrylate double bond is added by methanol (Figure 4). Azoxystrobin acid is the major product in the normal hydrolysis reaction.

**FIGURE 5:**
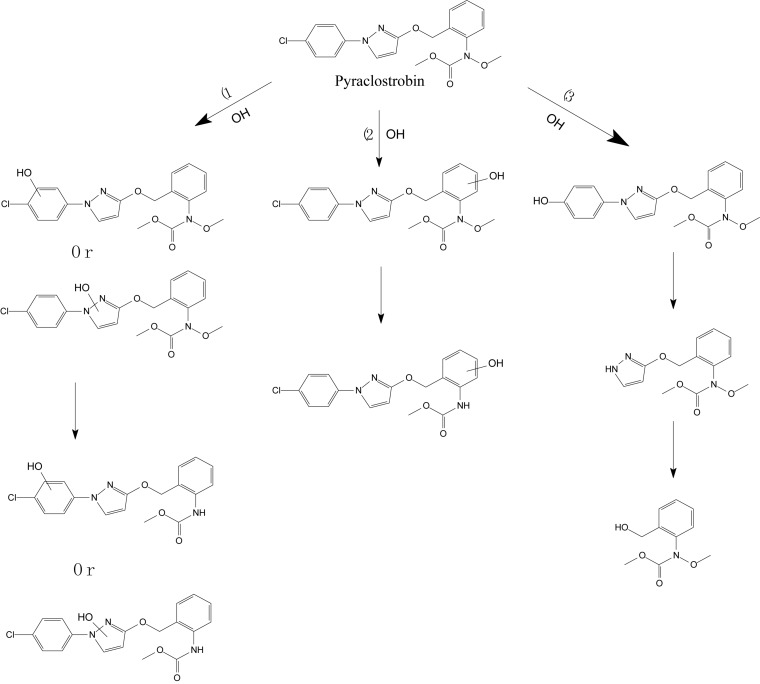
Proposed degradation pathway of pyraclostrobin via the photocatalysis reaction (based on Lagunas-Allué et al., 2012).

**FIGURE 6:**
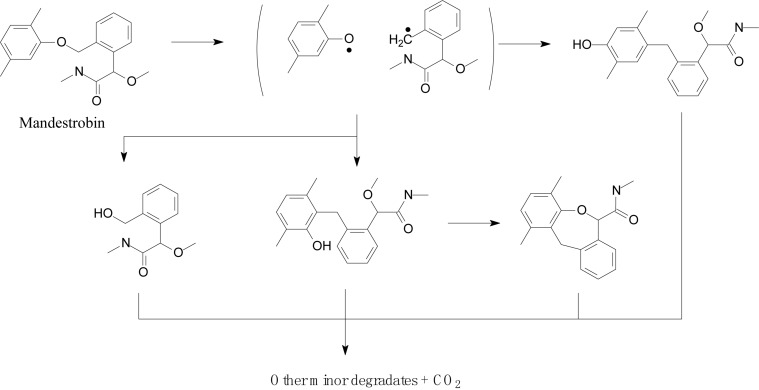
Proposed degradation pathway of mandestrobin (based on Adachi et al., 2018).

Lagunas-Allué et al. (2012) explored the photocatalytic degradation of pyraclostrobin in the presence of titanium dioxide (TiO_2_) as a photocatalyst and UV light irradiation. The photodegradation pathway included the rupture of the phenyl bond and pyrazol, scission of oxygen, substitution of a chloride atom by the hydroxyl group, and hydroxylation of aromatic rings (chloro-phenyl, phenyl, and pyrazol) followed by the loss of the *N*-methoxy group (Figure 5; Lagunas-Allué et al., 2012). Adachi et al. (2018) investigated the mandestrobin photodegradation process in synthetic humic water (SHW) and buffered aqueous solution under continuous irradiation of artificial sunlight (λ > 290 nm) (Figure 6). Homolytic bond cleavage at the benzyl phenyl ether of mandestrobin proceeded preferentially under direct photolysis, and photo-Claisen rearrangement products were formed by the subsequent recombination of geminate radicals in a solvent cage. They also reported that the formation of a benzyl alcohol derivative was enhanced by the photosensitized generation of hydroxyl radicals in water. The photolysis products were finally degraded and mineralized to carbon dioxide (Adachi et al., 2018).

**FIGURE 7:**
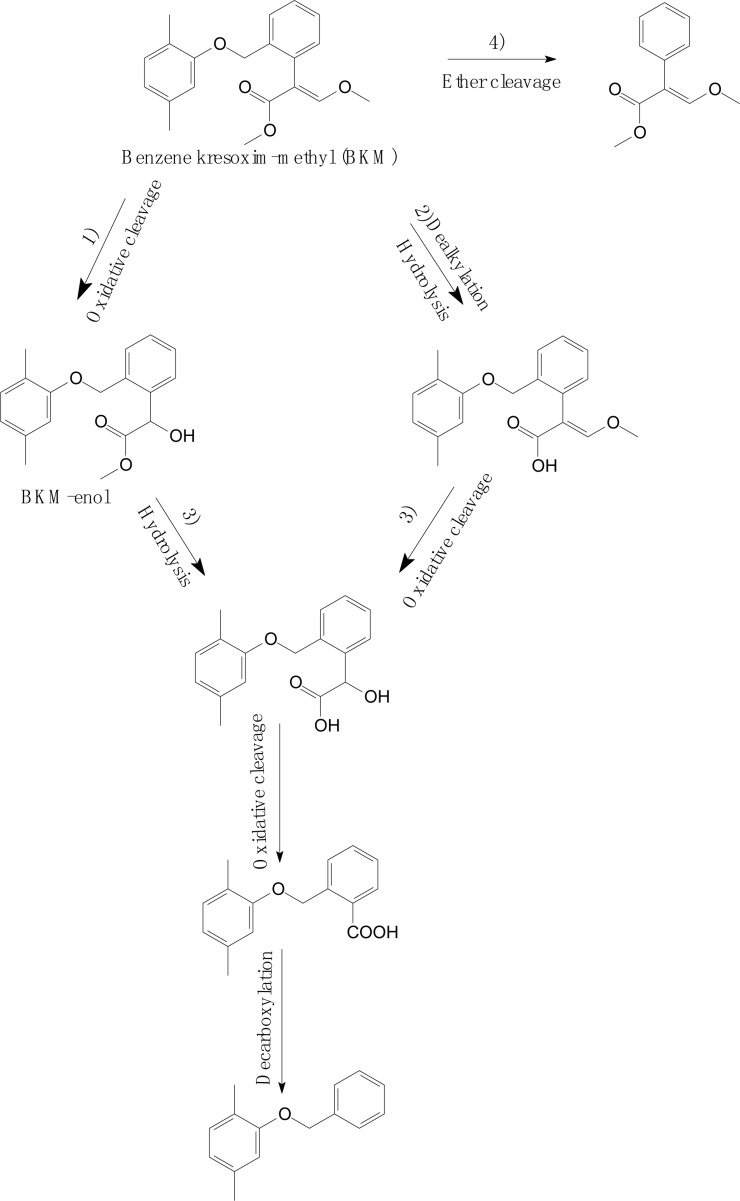
Proposed degradation pathways of benzene kresoxim-methyl (BKM) in aerobic soils (based on Wang et al., 2018).

Wang et al. (2018) investigated the degradation pathway of benzene kresoxim-methyl (BKM) in aerobic soils (Figure 7). As shown in Figure 7, the BKM degradation process occurs via multiple parallel reactions, including (1) oxidative cleavage of the acrylate double bond or hydroxylation to yield BKM-enol; (2) hydrolysis of the methyl ester or dealkylation; (3) further metabolism of the intermediates, such as oxidative cleavage and decarboxylation; and (4) cleavage of the ether linkage between dimethyl benzene and the phenylacrylate ring of BKM. Similarly, Romeh (2017) found that azoxystrobin also metabolized to azoxystrobin-enol in *Plantago major*. A similar degradation process was also discovered in trifloxystrobin, which forms a major metabolite of trifloxystrobin acid in plant materials (Liu et al., 2011). These results confirmed the basic degradation pathways of strobilurins that were identified and summarized by Balba (2007).

## Microbial Degradation of Strobilurins

Microbial degradation is considered as the most significant pathway for strobilurin removal (Bacmaga et al., 2015; Chen et al., 2018). Hitherto, several strobilurin-degrading microbes have been isolated and include *Bacillus*, *Pseudomonas*, *Klebsiella*, *Stenotrophomonas*, *Arthrobacter*, *Rhodanobacter*, *Cupriavidus*, and *Aphanoascus* (Lopes et al., 2010; Clinton et al., 2011; Howell et al., 2014; Bacmaga et al., 2015; Chen et al., 2018). Documented strobilurin-degrading strains are listed in Table 1. A *Klebsiella* strain 1805 isolated from soil presented valuable attributes for the degradation of pyraclostrobin and triazole fungicide epoxiconazole (Lopes et al., 2010). Clinton et al. (2011) reported that four different species (*Stenotrophomonas maltophilia*, *Bacillus amyloliquefaciens*, *Bacillus flexus*, and *Arthrobacter oxydans*) isolated from soil could use trifloxystrobin as a carbon source; however, they failed to isolate azoxystrobin-degrading bacteria. Howell et al. (2014) followed the sequential soil and liquid culture enrichment technique to isolate two bacterial strains (*Cupriavidus* sp. CCH2 and *Rhodanobacter* sp. CCH1) that use azoxystrobin as a sole carbon and nitrogen source. In the presence of an additional nitrogen source, both isolates also partially degraded other strobilurins, such as trifloxystrobin, pyraclostrobin and kresoxim-methyl. Four species of *Bacillus* spp. and two species of *Aphanoascus* spp. isolated from contaminated soil also survived on a high dose of azoxystrobin (22.50 mg⋅kg^–1^) (Bacmaga et al., 2015). In addition, two microbial communities (HI2 and HI6) capable of catabolizing pyraclostrobin as a sole carbon and nitrogen source were obtained from Hawaiian soils by Chen et al. (2018). Taxonomic identification showed that *Pseudomonas* was the dominant bacteria in both HI2 and HI6 microbial communities.

**TABLE 1:** Strobilurin-degrading microbes and microbial communities.

Strain	Source	Comments	References
*Klebsiella* sp. 1805	Soybean-grown soil after long-term use of Opera, Brazil	31.7% degradation of praclostrobin (108.3 μM) was achieved after 120 h 100% degradation of praclostrobin (36.5 μM) was achieved after 120 h	Lopes et al., 2010
*Stenotrophomonas maltophilia*	Strobilurin-contaminated soil, Australia	Initial trifloxystrobin concentration of 25 μg⋅L^–1^ Incubated at 28°C for 15 days	Clinton et al., 2011
*Arthrobacter oxydans*	Strobilurin-contaminated soil, Australia	Initial trifloxystrobin concentration of 25 μg⋅L^–1^ Incubated at 28°C for 15 days	Clinton et al., 2011
*Bacillus amyloliquefaciens*	Strobilurin-contaminated soil, Australia	Initial trifloxystrobin concentration of 25 μg⋅L^–1^ Incubated at 28°C for 15 days	Clinton et al., 2011
*Bacillus flexus*	Strobilurin-contaminated soil, Australia	Initial trifloxystrobin concentration of 25 μg⋅L^–1^ Incubated at 28°C for 15 days	Clinton et al., 2011
*Rhodanobacter* sp. CCH1	Soil received no pesticide applications, United Kingdom	88.5% degradation of azoxystrobin (25 mg⋅L^–1^) was achieved after 16 days with an additional source of nitrogen Utilized other strobilurins including trifloxystrobin pyraclostrobin and kresoxim-methyl	Howell et al., 2014
*Cupriavidus* sp. CCH2	Soil received no pesticide applications, United Kingdom	85.5% degradation of azoxystrobin (25 mg⋅L^–1^) was achieved after 16 days with an additional nitrogen source Utilized other strobilurins including trifloxystrobin pyraclostrobin and kresoxim-methyl	Howell et al., 2014
*Bacillus cereus* (KC848897.1)	Soil from Tomaszkowo near Olsztyn in northeastern Poland	Survived against highest dose of azoxystrobin (22.50 mg⋅kg^–1^)	Bacmaga et al., 2015
*Bacillus weihenstephanensis* (KF831381.1)	Soil from Tomaszkowo near Olsztyn in northeastern Poland	Survived against highest dose of azoxystrobin (22.50 mg⋅kg^–1^)	Bacmaga et al., 2015
*Bacillus* sp. (LM655314.1)	Soil from Tomaszkowo near Olsztyn in northeastern Poland	Survived against highest dose of azoxystrobin (22.50 mg⋅kg^–1^)	Bacmaga et al., 2015
*Bacillus megaterium* (KJ843149.1)	Soil from Tomaszkowo near Olsztyn in northeastern Poland	Survived against highest dose of azoxystrobin (22.50 mg⋅kg^–1^)	Bacmaga et al., 2015
*Aphanoascus terreus* (AB861677.1)	Soil from Tomaszkowo near Olsztyn in northeastern Poland	Survived against highest dose of azoxystrobin (22.50 mg⋅kg^–1^)	Bacmaga et al. (2015)
*Aphanoascus fulvescens* (JN943451.1)	Soil from Tomaszkowo near Olsztyn in northeastern Poland	Survived against highest dose of azoxystrobin (22.50 mg⋅kg^–1^)	Bacmaga et al., 2015
(Microbial communities HI2 and HI6)	Soil from University of Hawaii at Manoa, Honolulu, United States.	More than 93.8% degradation of pyraclostrobin (10 mg⋅L^–1^) was achieved after 3 days	Chen et al., 2018

Among strobilurin-degrading microbes, including bacteria and fungi, bacteria play the most critical role. The long-term application of strobilurins affects microbial counts and microbial biodiversity in ecosystems. Due to the unique mechanism of action, strobilurins may directly affect fungal biomass by inhibiting mitochondrial respiration, which can induce a shift from fungal to bacterial dominance in soil activities (Adetutu et al., 2008; Bacmaga et al., 2015). For instance, the significant biodegradation of the parent azoxystrobin occurs within 21 days, even in the absence of light, which can reduce fungal diversity in soil. This group of chemicals inhibited the growth and development of fungi, whereas bacterial diversity remained unaffected under the same conditions (Adetutu et al., 2008).

**FIGURE 8:**
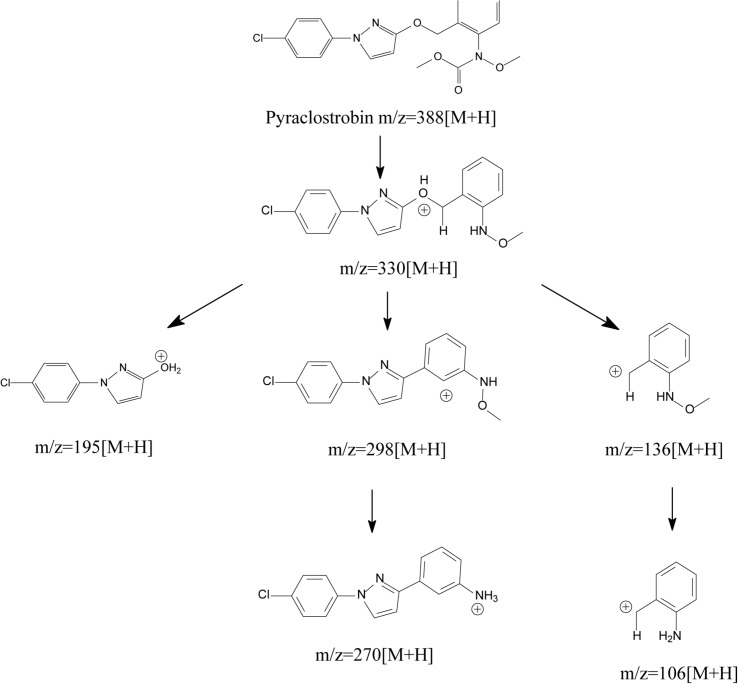
Proposed metabolic mechanisms of pyraclostrobin in microorganisms (based on Chen et al., 2018).

Generally, degrading enzymes, especially esterase, occupy an important place in the process of ester containing pesticide biodegradation (Chen et al., 2014, 2015; Zhan et al., 2018a, Zhan et al., 2020; Bhatt et al., 2019b, 2020). Chen et al. (2018) proposed the metabolic mechanism of pyraclostrobin biodegradation. As illustrated in Figure 8, pyraclostrobin detoxification was facilitated by carbamate hydrolysis, in which the tertiary amine group of pyraclostrobin was decarboxylated and hydrolyzed to the primary amine group. The metabolic mechanism also indicated that carboxylesterase plays a key role in pyraclostrobin biodegradation. Though the structures of strobilurins were complex due to many active sites on its molecular, the possible molecular mechanisms involved in strobilurin biodegradation pathways were similar (Balba, 2007; Chen et al., 2018; Wang et al., 2018). It was generally regarded that the carboxylester hydrolysis via esterase was the primary degradation mechanism of strobilurins in microorganisms (Balba, 2007; Chen et al., 2018). Hitherto, a few studies have focused on strobilurin-degrading enzymes. For instance, Katagi (2006) found that microbial degradation of azoxystrobin occurs by the hydrolysis of carboxyl ester bonds, indicating the central role of esterase in strobilurin degradation. Similarly, Chen et al. (2018) also inferred that carboxylesterase might be beneficial to the detoxification of pyraclostrobin. However, information regarding the degradation mechanisms of strobilurins is still insufficient. Previous studies observed that strobilurin metabolites were even more persistent and toxic in the environment than the parent compound (Ghosh and Singh, 2009; Khandelwal et al., 2014). Thus, deeper insights into the possible molecular mechanisms of these pesticides and their eventual fate are indispensable.

## Bioremediation Potential of Strobilurin-Degrading Microorganisms

The accumulation of toxic and carcinogenic environmental pollutants, such as pesticides, plastics, dyes and other hydrocarbon-containing substances, is hazardous to ecosystems (Wasilkowski et al., 2012; Morillo and Villaverde, 2017; Huang et al., 2019). Thus, the development of a quick, easily applied, socially acceptable and eco-friendly approach against these xenobiotic compounds, such as enzyme-based bioremediation, is necessary (Karigar and Rao, 2011; Liu et al., 2015; Sharma et al., 2018; Zhan et al., 2018b). Microorganisms have the potential to degrade the toxic pesticides easily through their metabolic pathways (Bhatt et al., 2019b). Various universal and specific metabolic pathways participated in strobilurin degradation. During biodegradation processes, pesticides induce the expression of genes and enzymes that are upregulated under stress conditions (Gangola et al., 2018; Bhatt et al., 2019a, b; Lin et al., 2020). Hence, it is crucial to isolate specialized microbial strains, as well as the efficient degrading enzymes and correlated genes, to accelerate the degradation rate and mineralization of strobilurins in contaminated soil/water environments.

Soil microbes exhibited bioremediation potential of strobilurin-contaminated environments. According to Liu et al. (2014), the degradation and mineralization process of a newly developed strobilurin fungicide (SYP-3343) was affected and accelerated by soil microbes. Zhao et al. (2017) explored the kinetic fate of BKM in aerobic soils and suggested that the soil type and microbial community composition controlled the process of BKM degradation and mineralization. Soil microbes can sharply decrease the residues of BKM as well. The persistence of strobilurins in soil varies from days to months at up to 50% degradation (DT50), depending on the soil microbial and chemical properties (British Crop, and Protection Council, 2009; Ghosh and Singh, 2009; EFSA, 2010). In addition, factors such as temperature, pH, initial strobilurin concentration, nutrients and additional carbon sources affect the biodegradation process in soil (Chen et al., 2012, 2013; Cycoń and Piotrowska-Seget, 2016). The most desirable candidates for strobilurin biodegradation are microorganisms that are active under various environmental conditions and can degrade a wide range of strobilurins. It is also necessary to study the complex behavior of microbial communication networks, which play a decisive role in the microbes’ adaptation to pollutants (Sharma et al., 2018). Azoxystrobin degrading potential of *Arthrobacter*, *Mycobacterium*, and *Rhodococcus* reported and correlated with increase and decrease of soil microbial community in combination of antibiotics. Metagenomics study suggested that some of the soil microbial genera significantly decreased (*Proteobacteria* and *Firmicutes*) whereas abundance of other like *Actinobacteria* increased in presence of strobilurins (Han et al., 2019). After soil applications strobilurins can reach the groundwater but the soil microorganisms have the potential to degrade them and reduce their effect in water system (Guo et al., 2017). Additionally, as the degradation of contaminants by microorganisms is a slow process, more details about strobilurin biodegradation pathways, degradation enzymes and genes that encode for key enzymes will help to identify the new bioremediation strategies.

## Conclusion and Future Perspectives

The intensive/large-scale application of strobilurins in agricultural fields has increased contamination of the surrounding soil/water environments. Strobilurin toxicity may result in ecosystem imbalance and food-web disruption. Strobilurin residues have increased in trophic level due to biomagnification; thus, the development of degradation technologies for these pesticides is necessary. Strobilurin phototransforms under sunlight via various mechanisms, and photochemical degradation is one of the most prevalent processes underlying strobilurin degradation in water. However, uncontrollable reaction conditions and inefficiency in cleaning the environment *in situ* make this process unsuitable for strobilurin residues. The microbial removal of strobilurin contaminations from the environment is an efficient and cost-effective alternative approach. Therefore, the potential of strobilurin-degrading microorganisms and their enzymes should be studied. However, published literature about the use of microorganisms to bioremediate a strobilurin-contaminated environment is still insufficient. In addition, the efficient strobilurin-degrading enzymes and correlated genes in microorganisms, have not yet been investigated. Studies on cooperative degradation activities by microbial communities can help prevent the accumulation of toxic metabolites during degradation. Therefore, detailed foundation work should be accomplished before the field application of strobilurin-degrading microorganisms is undertaken. The application of recently developed high throughput technologies for detecting strobilurin-degrading microbes in agricultural soil/water can generate better information about strobilurin degradation.

## Author Contributions

SC conceived of the presented idea. YF contributed to the writing and prepared the figures and tables. YH, HZ, PB, and SC participated in revising the manuscript. All authors approved it for publication.

## Conflict of Interest

The authors declare that the research was conducted in the absence of any commercial or financial relationships that could be construed as a potential conflict of interest.
